# Sex Hormones in Hemolymph of Red King Crabs from the Barents Sea

**DOI:** 10.3390/ani11072149

**Published:** 2021-07-20

**Authors:** Alexander G. Dvoretsky, Elena V. Tipisova, Aleksandra E. Elfimova, Viktoria A. Alikina, Vladimir G. Dvoretsky

**Affiliations:** 1 Murmansk Marine Biological Institute (MMBI), 183010 Murmansk, Russia; v-dvoretsky@yandex.ru; 2 N. Laverov Federal Center for Integrated Arctic Research of the Ural Branch of the Russian Academy of Sciences (FECIAR UrB RAS), 163000 Arkhangelsk, Russia; tipisova@rambler.ru (E.V.T.); a.elfimova86@mail.ru (A.E.E.); victoria-popcova@yandex.ru (V.A.A.)

**Keywords:** *Paralithodes camtschaticus*, red king crab, sex hormones, testosterone, 17β-estradiol, Barents Sea

## Abstract

**Simple Summary:**

Well-known sex hormones, testosterone and 17β-estradiol, play a crucial role in the reproduction of vertebrates. Biochemical assays have detected these substances in a few crustaceans, and it has been hypothesized that these hormones are involved in the regulation of crustacean reproduction. Red king crab is a large commercially important species harvested both in their native areas (North Pacific) and in the area of its introduction (Barents Sea). The presence of 17β-estradiol and testosterone and fluctuations of their concentrations in relation to different factors have not yet been investigated. For this reason, we provided a pilot study to reveal the levels of sex hormones in hemolymph of red king crabs captured in the coastal Barents Sea. These hormones were detected in the crabs and we compared our data with previously published data involving a wide range of crustaceans. We found seasonal variations in the level of testosterone with the maximum in the spawning period. Our data expand the current knowledge about the red king crab physiology and may be used for the development of its aquaculture.

**Abstract:**

The presence of vertebrate-related steroid sex hormones has been reported in both freshwater and marine crustaceans. However, despite the commercial importance of king crabs, many aspects of their endocrinology are still unknown. For this reason, we examined hemolymph samples of the red king crab *Paralithodes camtschaticus* from the Barents Sea population for the presence of testosterone and 17β-estradiol using radioimmunoassay. The mean testosterone concentration was 0.46 ± 0.04 (range 0.08–1.39) ng mL^–1^, whereas the mean 17β-estradiol concentration was 1248.9 ± 91.4 (range 217.7–4100.1) pg mL^–1^. In general, the levels of 17β-estradiol and testosterone in red king crabs were higher than reported for the hemolymph of amphipods, crabs, and shrimps from warm and temperate waters, probably because the king crabs analyzed were larger and heavier than the other crustaceans. The concentrations of sex steroids did not differ significantly between males and females and between immature and mature red king crabs. Seasonal variations in the level of testosterone with the maximum value in the spawning period (May) indicate a potential role of the sex hormones in the maturation and reproduction processes of red king crab. Taking into account the slow growth rate in *P. camtschaticus*, our data could be useful not only for further physiological studies but also for the development of reliable techniques for red king crab aquaculture.

## 1. Introduction

The red king crab, *Paralithodes camtschaticus* (Tilesius, 1815), is a large commercially important species native to the North Pacific. A new self-sustaining population of this crab was reported to be established in the Barents Sea in the mid-1990s after its successful introduction by Russian specialists in the 1960s. Exponential population growth and range expansion of the crab has led to an increase in *P. camtschaticus* stock indices. Commercial fisheries for the red king crab were opened in 2002 in Norway and 2004 in Russia [1,2]. In Russian waters of the Barents Sea, annual landings of red king crab accounted for 9187 t in 2018, 9836 t in 2019 and 10,820 t in 2020 [3,4].

In the Barents Sea, different aspects of the red king crab biology and ecology have been intensively studied by marine scientists due to the invasive status of *P. camtschaticus* and its economic importance. Russian researchers have focused on distribution patterns, migration activity, molting and growth, reproduction, symbiotic relationships and effects on local benthic communities [2,4,5,6,7,8,9,10]. However, our knowledge of the physiology of this introduced species in its new environment is scarce, and information on biochemical profiles of red king crab is needed. Recently, we have reported concentrations of circulation ecdysteroids in hemolymph of *P. camtschaticus* [11]. Concentrations of a respiratory pigment, hemocyanin, were studied as a function of the meat content of commercial red king crabs from the Barents Sea [12].

The use of sex hormones and other hormones to regulate biological processes is a common strategy of vertebrate animals [13,14,15,16,17]. Usually, alterations in steroid hormone metabolism relate with effects on steroid hormone-dependent processes such as reproduction and growth [18]. Such alterations may be used as a biomarker for the effects of endocrine disruptors in invertebrates. Most stages of the steroidogenic pathways discovered for vertebrates have been demonstrated to occur in invertebrates. Although it was proposed that crustaceans do not actively synthesize vertebrate-type steroids in the same ways that echinoderms and mollusks do [19], the metabolism of these steroids was only investigated in a few crustaceans [18,20]. In crabs and lobsters, the endogenous precursor androstenedione is reduced to testosterone [21], while estrone is converted to estradiol [20]. Sex steroid-like substances can also be synthesized from cholesterol [22].

A high similarity was observed among the endocrine system of different crustaceans [23,24] and, therefore, sex hormones similar to those in vertebrates have been identified in several crustacean species including amphipods, shrimps, crayfish, lobsters, and crabs [19,25,26,27,28]. The presence of these hormones and their concentrations in red king crabs both in their native and non-native areas have not yet been reported. For this reason, the aim of our study was to measure concentrations of vertebrate-type sex hormones in hemolymph of the king crabs *P. camtschaticus* from the Barents Sea in relation to their sex and age.

## 2. Materials and Methods

**Figure 1:**
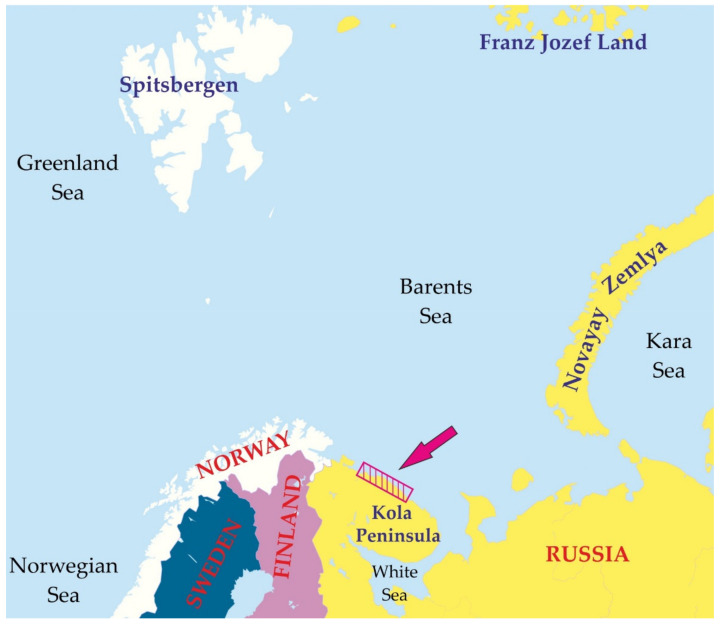
Study area in the southern Barents Sea.

Red king crabs were collected in August 2008 and May, July, and October 2009 in the coastal zone of the Barents Sea (Figure 1). The crabs were collected by divers at 5–40 m depths and using bottom traps at 50–60 m depths. Sex of each crab was determined visually. The crabs were weighed and their sizes were measured across the length of the carapace (CL, the greatest straight-line distance from the posterior margin of the right eye orbit to the medial-posterior margin of the carapace) [29]. All the crabs used for biochemical analyzes had new or old shells (2–24 months post ecdysis) [29]. Male crabs with CL < 90 mm were considered “immature,” whereas males with the CL > 90 mm were classified as “mature” according to Dvoretsky and Dvoretsky [8]. This separation is supported by the results of histological studies: in our samples the crabs smaller than 90 mm CL had no ripe spermatozoa in their testes in contrast to larger specimens. The same female groups were separated according to both histological data (immature females had oocyte diameter < 280 μm and white ovaries [5]) and size-at-maturity data (absence or presence of external eggs on their pleopods) [9]. In total, 23 immature (7 males and 16 females) and 33 mature (26 males and 7 females) crabs were analyzed.

After measuring the crabs, we sampled their hemolymph (0.6 mL) from the articulating membrane between the dactyl and propodus of the third walking leg using a sterile syringe with a needle, both washed with an anticoagulant containing EDTA and sodium citrate to prevent coagulation of the samples [29]. Each hemolymph sample was transferred to a sterile 2-mL plastic tube containing 1.4 mL of 96% ethanol. The samples were frozen and then transferred to the laboratory of the Institute of Physiology of Natural Adaptations (Arkhangelsk, Russia) for biochemical analyses.

Radioimmunoassays were carried out according to the protocols suggested by the manufacturer (A Beckman Coulter Company, Villepinte, France) using Immunotech RIA Testosterone or Immunotech RIA Estradiol test kits. In the case of testosterone, a 50-μL hemolymph sample and 500 μL of ^125^I-labelled testosterone were added serially to an antibody-coated tube. In the case of 17β-estradiol, a 100-μL hemolymph sample was used in the assays. A tube containing 500 μL of ^125^I-labelled testosterone (or estradiol) was included in every assay to determine the total ^125^I-activity (the sample “T”). The “testosterone” tubes were incubated at 37 °C in a waterbath for 3 h while the “estradiol” tubes were incubated with constant shaking (350 rpm) at ambient temperature for 3 h. At the end of incubation, the radioactivity of samples was determined using a scintillation counter (ARIAN, producer OOO VITAKO, Moscow, Russia). The standard graphs were plotted and the concentrations of testosterone and 17β-estradiol were obtained from the standard plot. The results were multiplied by a dilution factor of 3.33. The analytical sensitivity of the testosterone kit was 0.025 ng mL^–1^, the working RIA range was 0.025−20 ng mL^–1^, the coefficient of variation between samples was 14.8%. For the estradiol kit, these levels were <6 pg mL^–1^, 6−5000 pg mL^–1^ and 12.1%, respectively.

Carapace lengths and weight data in male and female red king crabs were compared using a one-way analysis of variance (ANOVA) or non-parametric Kruskal–Wallis tests in the case of non-normal data distribution. Since the sex hormone data were not normally distributed and failed testing for homogeneity of variances (modified Levene’s test, *p* < 0.05), testosterone and 17β-estradiol concentrations in relation to crab sex, crab size, and sampling season were compared using the Kruskal–Wallis test. When the test was significant, medians for different groups were compared using Bonferroni tests and differences were considered significant if *p* < 0.05. Statistical analyzes were carried out using STATISTICA (data analysis software system; http://www.statsoft.com/, accessed on 10 May 2020), version 6. Data are presented as means ± SE (standard error).

The taxonomic nomenclature follows the nomenclature according to WoRMS [30].

## 3. Results

**Figure 2:**
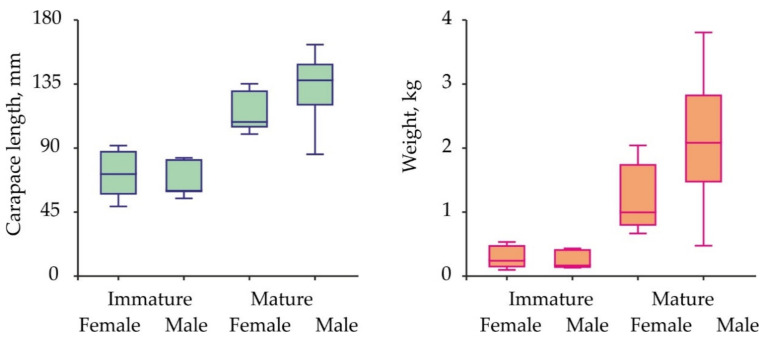
Carapace length and weight variations in the Barents Sea red king crabs analyzed for sex hormones.

Size and weight variations in red king crabs collected for the hemolymph sampling are presented in Figure 2.

Carapace length ranged from 49.0 to 162.7 mm, body weight—from 97 to 3805 g. Mean carapace length in immature male and female crabs was similar (Kruskal-Wallis test, df = 1, H = 0.090, *p* = 0.764). The same result was obtained for mature specimens (Kruskal-Wallis test, df = 1, H = 3.421, *p* = 0.064). Mean weight did not differ significantly between immature males and females (ANOVA, df = 1, F = 0.600, *p* = 0.447). Mature males were heavier than mature females (ANOVA, df = 1, F = 4.645, *p* = 0.039).

**Figure 3:**
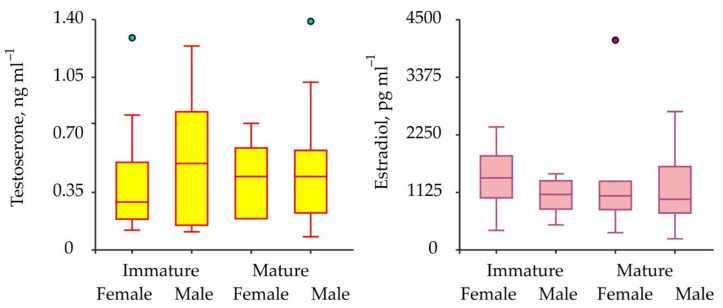
Variations of sex hormones in immature and mature red king crabs from the coastal zone of the Barents Sea. Green and red circles are outliers.

Biochemical assays have shown the presence of both testosterone and 17β-estradiol in the hemolymph of the crabs analyzed. Concentrations of testosterone varied from 0.08 to 1.39 ng mL^–1^ (mean for combined male and female data 0.46 ± 0.04 ng mL^–1^), while concentrations of 17β-estradiol ranged from 217.7–4100.1 pg mL^–1^ (1248.9 ± 91.4 pg mL^–1^) (Figure 3).

**Figure 4:**
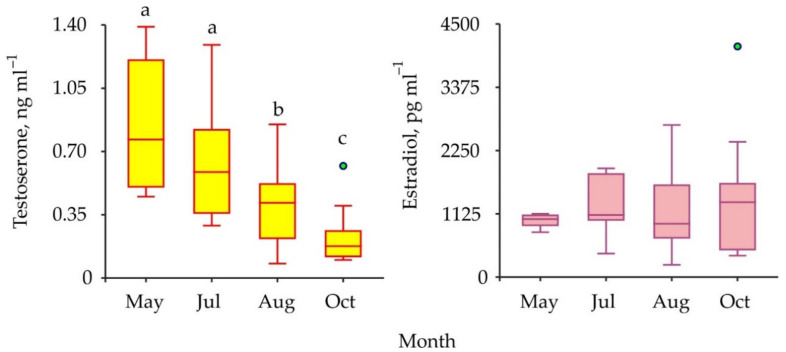
Seasonal variations in vertebrate-type sex hormones in hemolymph of red king crabs from the Barents Sea. Green circles are outliers. Bars with the same letter are not significantly different (*p* > 0.05).

The comparisons of sex steroids in male and female red king crabs as well as in immature and mature specimens have shown that concentrations of both testosterone and 17β-estradiol were similar between all the combinations of the groups compared (Table 1). To increase the power of further analysis we used pooled data from males and females at different maturational stages in one complex. Seasonal variations in testosterone and 17β-estradiol concentrations are presented in Figure 4.

The mean level of testosterone decreased significantly from May to October whereas there was no significant difference detected among the seasonal concentrations of 17β-estradiol in the hemolymph samples (Table 1).

## 4. Discussion

According to our published [8] and unpublished size-at-age data, the age of the red king crabs used in this study varied from 3 to 11 years (females) and 3 to 13 years (males). The mean weight of large males was higher than that calculated for females because mature male red king crabs invest more energy in somatic growth in comparison to females, which invest the major proportion of their resources into reproduction processes [7,10].

Our study indicated that red king crabs have detectable concentrations of 17β-estradiol and testosterone in both male and female hemolymph. In general, the level of 17β-estradiol found for the female red king crabs was higher (Table 2) in comparison to females of some other decapod species such as Norwegian lobster *Nephrops norvegicus* in western Scotland [31], mud crabs *Scylla serrata* in India [32] and *Scylla paramamosain* in China [27], Chinese mitten crab *Eriocheir sinensis* in China [33], edible crab *Spiralothelphusa senex* (cited as *Oziothelphusa senex senex*) in India [34], soldier crab *Mictyris brevidactylus* in northern Taiwan [35] and grass shrimp *Pandalus kessleri* in Japan [25].

The estradiol concentration registered in the red king crab male hemolymph was higher than in *Eriocheir sinensis* males [33], while the male testosterone level was comparable to the concentration reported for the hemolymph of *S. paramamosain* [27]. In the red king crabs, the levels of both hormones were much higher than in gammarid amphipods *Gammarus duebeni celticus* and *G. pulex* in Northern Ireland and *G. pseudolimnaeus* in Waukesha County, WI, USA [28]. It is difficult to evaluate causes responsible for this result, but most likely the higher concentrations of sex hormones in the red king crab hemolymph were associated with their larger size and weight. For example, the mud crabs *S. paramamosain* used in the study described by Huiyang et al. [27] had a body length of 36–87 mm, while the average weight of mature *Spiralothelphusa senex* females used for biochemical assays in the study by Swetha et al. [34] was 32–37 g. Additionally, the largest CL reported for adult female *Nephrops norvegicus* is about 50 mm CL [41]; females of *Scylla serrata* attain sexual maturity after reaching 80 mm carapace width (CW) [42], and females of *Eriocheir sinensis* become mature at 34.1 ± 3.9 mm CL with a weight of 26.6 ± 8.1 g [43]. The largest adult females of *Mictyris brevidactylus* belong to a size class of 110–120 mm CW [44]. The majority of the mentioned crustaceans occur in temperate and warm waters whereas the red king crabs live in a cold-water environment. Different habitats may also contribute to differences between the levels of sex hormones in *P. camtschaticus* and those crustacean species.

In vertebrates, both males and females produce testosterone and 17β-estradiol, although the concentrations of these hormones may vary significantly depending on sex and the reproductive cycle [28].

Taking into account the differences in invertebrate and vertebrate endocrine systems, there was no reason to assume that typical sex-specific differences in sex hormones among vertebrates would also be found in red king crabs. Our data support to some extent this assumption: we found no significant differences in testosterone and 17β-estradiol concentrations in relation to age and sex of the red king crabs. Similar results were reported by other authors. For example, males of *Gammarus duebeni celticus*, *G. pulex,* and *G. pseudolimnaeus* had comparable concentrations of 17β-estradiol, and females of these species had comparable concentrations of testosterone [28].

On the other hand, the ovaries of some decapods may synthesize 17β-estradiol and release this hormone into the hemolymph, and then it may reach the hepatopancreas to stimulate vitellogenin synthesis [26]. For this reason, concentrations of sex steroids in gonads, eggs, and hepatopancreas of crustaceans are higher than in their hemolymph (Table 2). Thus, an association between sex hormone profiles and stages of vitellogenesis was detected in some decapod species [45]. For example, the levels of 17β-estradiol increased significantly in the tissues of crabs *Scylla serrata, S. paramamosain*, and *Spiralothelphusa senex* during late vitellogenesis [27,32,34]. Similar trends were reported for testosterone as well [27]. In addition, concentrations of sex hormones were found to positively correlate with the reproductive status of decapod crustaceans, as they were high during the pre-reproductive season and low during the non-reproductive season [35,37,46]. We also found seasonal changes in the testosterone profile assayed in the Barents Sea red king crabs. The maximum (0.8 ng mL^–1^) was registered in May and the minimum (0.2 ng mL^–1^) in October. Spawning season in *P. camtschaticus* lasts from February to May with a peak in April [5]. Thus, we expected to find the highest level of testosterone in the May samples of adult crabs. We found no seasonal fluctuations in the 17β-estradiol concentration. This result could be explained by the fact that we studied hemolymph, whereas estrogens are synthesized in the ovaries of red king crabs. These organs are much heavier than the male testes. Monthly variations of testosterone in immature crabs more likely reflected seasonal patterns of *P. camtschaticus* maturation in the Barents Sea.

The effects of 17β-estradiol on vitellogenesis (vitellogenin synthesis, oocyte development, an increase of ovarian indices, and growth rates) have been shown for the kuruma prawn *Marsupenaeus japonicus* [47], giant freshwater prawn *Macrobrachium rosenbergii* [48], freshwater crayfish *Cherax albidus* [49], burrowing crab *Chasmagnathus grdnulata* [50], and lobsters *Homarus americanus* and *Panulirus homarus* [51]. In addition, Nagabhushanam and Kulkarni [52] showed that exogenous testosterone can stimulate testicular development in the marine penaeid prawn, *Mierspenaeopsis hardwickii* (cited as *Parapenaeopsis hardwickii*). This hormone also induced sperm development and subsequent mating success in male tiger shrimps *Penaeus monodon* [53]. In contrast, the results by Okumura and Sakiyama [38] suggested that 17β-estradiol and testosterone did not play an important role in ovarian development in *Penaeus japonicus* (cited as *Marsupenaeus japonicus*), and Koskela et al. [54] reported no effect 17β-estradiol on development and reproduction of the tiger prawn *Penaeus esculentus*.

In the last several decades, aquaculture has grown rapidly in complexity and in volume around the world. The majority of aquaculture businesses require substantial amounts of both operating and investment capital. For this reason, profitable aquaculture cannot be organized without excellent knowledge on the biology and ecology of target species [55,56,57]. The development of specific methods to increase growth rates of slow-growing species such as red king crab is a great challenge for modern science [58,59], and our results may have important implications for further studies focused on aquaculture of *P. camtschaticus*.

## 5. Conclusions

Because molting and reproduction in crustaceans are controlled both by ecdysteroids (molting hormones) and vertebrate-like steroids, these hormones are considered as potential inductors of growth and maturation processes in cultured crustaceans. Understanding the controlling mechanisms of reproductive processes and molting in red king crabs is an important issue because of the slow growth rate of this species (the commercial size of 150 mm CW is reached at 10 years). Thus, the determination of the sex steroid levels in the red king crab *Paralithodes camtschaticus* is a first step toward developing new aquaculture techniques. The level of testosterone/17β-estradiol varied from 0.08–1.39 ng mL^–1^/217.7–2703.2 pg mL^–1^ in males and from 0.12–1.29 ng mL^–1^/335.6–4100.1 pg mL^–1^ in females. Further studies should reveal the possibility of the use of artificially synthesized hormonal preparations for accelerating the maturation process in red king crabs and, consequently, increasing their growth rates under laboratory conditions. Such studies will provide a baseline for the effective planning of regulatory measures and aquaculture programs in the Barents Sea region.

## Figures and Tables

**Table 1:** Results of Kruskal–Wallis tests comparing the differences in sex hormone levels of red king crabs.

Hormone	Comparisons	d	H	*p*
Testosterone	M-im vs. M-mat	1	0.436	0.509
	F-im vs. F-mat	1	0.875	0.349
	M-tot vs. F-tot	1	0.750	0.386
	May vs. July vs. August vs. October	3	24.030	0.00002
17β-estradiol	M-im vs. M-mat	1	0.001	0.982
	F-im vs. F-mat	1	0.698	0.404
	M-tot vs. F-tot	1	1.032	0.310
	May vs. July vs. August vs. October	3	0.950	0.813

Note. M—male, F- female, im—immature, mat—mature, tot—total, d—degree of freedom, H—chi-square level, *p*—probability level.

**Table 2:** Concentrations of vertebrate-like sex steroids identified in various crustacean species.

Steroid	Species	Sex	Matrix	Levels	Reference
E	*Nephrops norvegicus*	F	Eggs	900 pg g^–1^	[31]
E	*Nephrops norvegicus*	F	Hemolymph	800 pg mL^–1^	[31]
E	*Scylla serrata*	F	Hemolymph	230–1040 pg g^–1^	[32]
E	*Scylla serrata*	F	Ovary	180–1250 pg g^–1^	[32]
E	*Scylla serrata*	F	Hepatopancreas	300–4100 pg mg^–1^	[32]
E	*Scylla olivacea*	F	Hemolymph	970–3520 pg mL^–1^	[36]
E	*Pandalus latirostris*	F	Hemolymph	18.1–54.3 pg mL^–1^	[25]
E	*Penaeus monodon*	F	Hemolymph	30.4 pg mL^–1^	[37]
E	*Penaeus monodon*	F	Ovary	30–77 pg g^–1^	[37]
E	*Penaeus monodon*	F	Hepatopancreas	20–752.6 pg mg^–1^	[37]
E	*Penaeus japonicus*	F	Hemolymph	2.8–8.7 pg mL^–1^	[38]
E	*Gammarus duebeni*	F	Hemolymph	86.6 pg mL^–1^	[28]
E	*Gammarus duebeni*	M	Hemolymph	41.6 pg mL^–1^	[28]
E	*Gammarus pulex*	F	Hemolymph	38.8 pg mL^–1^	[28]
E	*Gammarus pulex*	M	Hemolymph	47.4 pg mL^–1^	[28]
E	*Gammarus pseudolimnaeus*	F	Hemolymph	18.8 pg mL^–1^	[28]
E	*Gammarus pseudolimnaeus*	M	Hemolymph	29.3 pg mL^–1^	[28]
E	*Scylla paramamosain*	F	Hemolymph	18.5–75.4 pg mL^–1^	[27]
E	*Emertia emeritus*	F	Ovary	2000–8000 pg g^–1^	[26]
E	*Emertia emeritus*	F	Hepatopancreas	2900–9700 pg g^–1^	[26]
E	*Macrobrachium rosenbergii*	F	Ovary	67–341 pg g^–1^	[26]
E	*Macrobrachium rosenbergii*	F	Hepatopancreas	168–663 pg g^–1^	[26]
E	*Eriocheir sinensis*	F	Hemolymph	118.8–291.8 pg mL^–1^	[33]
E	*Eriocheir sinensis*	F	Ovary	232.2–257.8 pg g^–1^	[33]
E	*Eriocheir sinensis*	M	Hemolymph	122.7–237.6 pg mL^–1^	[33]
E	*Eriocheir sinensis*	M	Testes	128.5–150.0 pg g^–1^	[33]
E	*Spiralothelphusa senex*	F	Hemolymph	27.9–134.2 pg mL^–1^	[34]
E	*Mictyris brevidactylus*	F	Hemolymph	0.2–0.6 pg g^–1^	[35]
E	*Mictyris brevidactylus*	F	Ovary	8.8–70.0 pg g^–1^	[35]
E	*Mictyris brevidactylus*	F	Hepatopancreas	0.6–122.9 pg g^–1^	[35]
T	*Nephrops norvegicus*	F	Ovary	19000 pg g^–1^	[31]
T	*Homarus americanus*	M	Testes	14300 pg g^–1^	[39]
T	*Gammarus duebeni*	F	Hemolymph	25.3 pg mL^–1^	[28]
T	*Gammarus duebeni*	M	Hemolymph	15.6 pg mL^–1^	[28]
T	*Gammarus pulex*	F	Hemolymph	22.1 pg mL^–1^	[28]
T	*Gammarus pulex*	M	Hemolymph	22.7 pg mL^–1^	[28]
T	*Gammarus pseudolimnaeus*	F	Hemolymph	21.7 pg mL^–1^	[28]
T	*Gammarus pseudolimnaeus*	M	Hemolymph	31.4 pg mL^–1^	[28]
T	*Scylla paramamosain*	M	Hemolymph	394–607 pg mL^–1^	[27]
T	*Penaeus japonicus*	F	Hemolymph	2.2–4.4 pg mL^–1^	[38]
T	*Neocaridina denticulata*	ND	Hemolymph	2700 pg g^–1^	[40]

Note: M—male, F—female, ND—no data, E—17β-estradiol, T—testosterone.

## Data Availability

Data will be made available upon any reasonable request to the corresponding author.

## References

[1] Dvoretsky A.G., Dvoretsky V.G. (2015). Commercial fish and shellfish in the Barents Sea: Have introduced crab species affected the population trajectories of commercial fish?. Rev. Fish Biol. Fish..

[2] Dvoretsky A.G., Dvoretsky V.G. (2018). Red king crab (*Paralithodes camtschaticus*) fisheries in Russian waters: Historical review and present status. Rev. Fish Biol. Fish..

[3] Dvoretsky A.G., Bichkaeva F.A., Baranova N.F., Dvoretsky V.G. (2021). Fatty acid composition of the Barents Sea red king crab (*Paralithodes camtschaticus*) leg meat. J. Food Compos. Anal..

[4] Dvoretsky A.G., Dvoretsky V.G. (2021). New echinoderm-crab epibiotic associations from the coastal Barents Sea. Animals.

[5] Kuzmin S.A., Gudimova E.N. (2002). Introduction of the Kamchatka (Red King) Crab in the Barents Sea. Pecularities of Biology, Perspectives of Fishery.

[6] Pavlova L.V., Matishov G.G. (2008). Red king crab trophic relations and its influence on bottom biocenoses. Biology and Physiology of the Red King Crab from the Coastal Zone of the Barents Sea.

[7] Dvoretsky A.G., Dvoretsky V.G. (2013). Population dynamics of the invasive lithodid crab, *Paralithodes camtschaticus*, in a typical bay of the Barents Sea. ICES J. Mar. Sci..

[8] Dvoretsky A.G., Dvoretsky V.G. (2014). Size-at-age of juvenile red king crab (*Paralithodes camtschaticus*) in the coastal Barents Sea. Cah. Biol. Mar..

[9] Dvoretsky A.G., Dvoretsky V.G. (2015). Size at maturity of female red king crab, *Paralithodes camtschaticus*, from the costal zone of Kola Peninsula (southern Barents Sea). Cah. Biol. Mar..

[10] Dvoretsky A.G., Dvoretsky V.G. (2020). Effects of environmental factors on the abundance, biomass, and individual weight of juvenile red king crabs in the Barents Sea. Front. Mar. Sci..

[11] Dvoretsky A.G., Dvoretsky V.G. (2010). Hemolymph molting hormone concentrations in red king crabs from the Barents Sea. Polar Biol..

[12] Moiseeva S.A., Moiseev S.I. (2008). Relation between muscular tissue condition in limbs and hemocyanin concentration in the hemolymph of red king crab (*Paralithodes camtschaticus*) in the Barents Sea. Probl. Fish..

[13] Kutzler M.A. (2020). Possible relationship between long-term adverse health effects of gonad-removing surgical sterilization and luteinizing hormone in dogs. Animals.

[14] Am-in N., Suwimonteerabutr J., Kirkwood R.N. (2020). Serum anti-mullerian hormone and estradiol concentrations in gilts and their age at puberty. Animals.

[15] Hyndman T.H., Algar K.L., Woodward A.P., Coiacetto F., Hampton J.O., Nickels D., Hamilton N., Barnes A., Algar D. (2020). Estradiol-17β pharmacokinetics and histological assessment of the ovaries and uterine horns following intramuscular administration of estradiol cypionate in feral cats. Animals.

[16] Massoud D., Lao-Pérez M., Ortega E., Burgos M., Jiménez R., Barrionuevo F.J. (2021). Divergent seasonal reproductive patterns in syntopic populations of two murine species in southern Spain, *Mus spretus* and *Apodemus sylvaticus*. Animals.

[17] García M.-L., Muelas R., Argente M.-J., Peiró R. (2021). Relationship between prenatal characteristics and body condition and endocrine profile in rabbits. Animals.

[18] Verslycke T., De Wasch K., De Brabander H.F., Janssen C.R. (2002). Testosterone metabolism in the estuarine mysid *Neomysis integer* (Crustacea; Mysidacea): Identification of testosterone metabolites and endogenous vertebrate-type steroids. Gen. Comp. Endocrinol..

[19] Janer G., Porte C. (2007). Sex steroids and potential mechanisms of non-genomic endocrine disruption in invertebrates. Ecotoxicology.

[20] Swevers L., Lambert J.G., De Loof A. (1991). Metabolism of vertebrate-type steroids by tissues of three crustacean species. Comp. Biochem. Physiol..

[21] Blanchet M.-F., Ozon R., Meusy J.J. (1972). Metabolism of steroids, *in vitro*, in the male crab *Carcinus maenas* Linne. Comp. Biochem. Physiol..

[22] Shih J.T., Liao C.F. (1998). Conversion of cholesterol to sex steroid-like substances by tissues of *Mictyris brevidactylus* in vitro. Zool. Stud..

[23] Charmantier G., Charmantier-Daures M., Van Herp F., Fingerman M., Nagabhushanam R. (1997). Hormonal regulation of growth and reproduction in crustaceans. Recent Advances in Marine Biotechnology.

[24] Swetha C.H., Sainath S.B., Reddy P.R., Reddy P.S. (2011). Reproductive Endocrinology of Female Crustaceans: Perspective and Prospective. J. Mar. Sci. Res. Dev..

[25] Quinitio E.T., Yamauchi K., Hara A., Fuji A. (1991). Profiles of progesterone-and estradiol-like substances in the hemolymph of female *Pandalus kessleri* during an annual reproductive cycle. Gen. Comp. Endocrinol..

[26] Gunamalai V., Kirubagaran R., Subramoniam T. (2006). Vertebrate steroids and the control of female reproduction in two decapods, *Emertia asiatica* and *Macrobrachium rosenbergii*. Curr. Sci..

[27] Huiyang H., Haihui Y., Shizhao H., Guizhong W. (2009). Profiles of gonadotropins and steroid hormone-like substances in the hemolymph of mud crab *Scylla paramamosain* during the reproduction cycle. Mar. Freshw. Behav. Physiol..

[28] Lewis S.E., Freund J.G., Riddell G.E., Wankowski J.L., Dick J.T.A., Baldridge M.G. (2015). Interspecific comparison of estrogen and testosterone concentrations in three species of amphipods (*Gammarus duebeni celticus*, *G. pseudolimnaeus*, and *G. pulex*). J. Crustacean Biol..

[29] Donaldson W.E., Byersdorfer S.E. (2005). Biological Field Techniques for Lithodid Crabs. Fairbanks, Alaska Sea Grant College Program.

[30] WoRMS (2021). World Register of Marine Species. http://www.marinespecies.org/.

[31] Fairs N.J., Evershed R.P., Quinlan P.T., Goad L.J. (1989). Detection of unconjugated and conjugated steroids in the ovary, eggs, and haemolymph of the decapod crustacean *Nephrops norvegicus*. Gen. Comp. Endocrinol..

[32] Warrier S.R., Tirumalai R., Subramoniam T. (2001). Occurrence of vertebrate steroids, estradiol 17 β and progesterone in the reproducing females of the mud crab *Scylla serrata*. Comp. Biochem. Physiol. Part A.

[33] Wei W., Wei H., Liu Q. (2005). Effect of estradiol in hemolymph and gonad on precociousness of *Eriocheir sinensis*. J. Fish. China.

[34] Swetha C.H., Girish B.P., Reddy P.S. (2016). Elucidation of the role of estradiol and progesterone in regulating reproduction in the edible crab, *Oziothelphusa senex senex*. RSC Adv..

[35] Shih J.-T. (1997). Sex steroid-like substances in the ovaries, hepatopancreases, and body fluid of female *Mictyris brevidactylus*. Zool. Stud..

[36] Amin-Safwan A., Muhd-Farouk H., Mardhiyyah M.P., Nadirah M., Ikhwanuddin M. (2019). Does water salinity affect the level of 17β-estradiol and ovarian physiology of orange mud crab, *Scylla olivacea* (Herbst, 1796) in captivity?. J. King Saud. Univ. Sci..

[37] Quinitio E.T., Hara A., Yamauchi K., Nakao S. (1994). Changes in the steroid hormone and vitellogenin levels during the gametogenic cycle of the giant tiger shrimp, *Penaeus monodon*. Comp. Biochem. Physiol..

[38] Okumura T., Sakiyama K. (2004). Hemolymph levels of vertebrate-type steroid hormones in female kuruma prawn *Marsupenaeus japonicus* (Crustacea: Decapoda: Penaeidae) during natural reproductive cycle and induced ovarian development by eyestalk ablation. Fish. Sci..

[39] Burns B.G., Sangalang G.B., Freeman H.C., McMenemy M. (1984). Isolation and identification of testosterone from the serum and testes of the American lobster (*Homarus americanus*). Gen. Comp. Endocrinol..

[40] Huang D.-J., Chen H.-C. (2004). Effects of chlordane and lindane on testosterone and vitellogenin levels in green neon shrimp (*Neocaridina denticulata*). Int. J. Toxicol..

[41] Tuck I.D., Atkinson R.J.A., Chapman C.J. (2000). Population biology of the Norway lobster, *Nephrops norvegicus* (L.) in the Firth of Clyde, Scotland II: Fecundity and size at onset of sexual maturity. ICES J. Mar. Sci..

[42] Prasad P.N., Neelakantan B. (1989). Maturity and breeding of the mud crab, *Scylla serrata* (Forskal) (Decapoda: Brachyura: Portunidae). Proc. Indian Acad. Sci. Anim. Sci..

[43] Tanglin Z., Zhongjie L., Yibo C. (2001). Survival, growth, sex ratio, and maturity of the Chinese mitten crab (*Eriocheir sinensis*) reared in a Chinese pond. J. Freshw. Ecol..

[44] Takeda S. (2005). Sexual differences in behaviour during the breeding seasonin the soldier crab (*Mictyris brevidactylus*). J. Zool. Lond..

[45] Subramoniam T. (2017). Steroidal control of vitellogenesis in Crustacea: A new understanding for improving shrimp hatchery production. Proc. Indian Acad. Sci. Anim. Sci..

[46] Pan J., Liu M., Chen T., Yongxu C., Xugan W. (2018). Immunolocalization and changes of 17beta-estradiol during ovarian development of Chinese mitten crab *Eriocheir Sinensis*. Cell Tissue Res..

[47] Yano I., Hoshino R. (2006). Effects of 17β-estradiol on the vitellogenin synthesis and oocyte development in the ovary of kuruma prawn (*Marsupenaeus japonicus*). Comp. Biochem. Physiol. Part A.

[48] Pakdeenarong N. (2019). Effect of estradiol-17β on embryonic tolerance, growth, and muscular compactness of giant freshwater prawn, *Macrobrachium rosenbergii*. J. Appl. Biol. Biotechnol..

[49] Coccia E., De Lisa E., Di Cristo C., Di Cosmo A., Paolucci M. (2010). Effects of estradiol and progesterone on the reproduction of the freshwater crayfish *Cherax albidus*. Biol. Bull..

[50] Zapata V., Lopez Greco L.S., Medesani D., Rodriguez E.M. (2003). Ovarian growth in the crab, *Chasmagnathus granulata* induced by hormones and neuroregulators throughout the year. In vivo and in vitro studies. Aquaculture.

[51] Subramoniam T., Kirubagaran R. (2010). Endocrine regulation of vitellogenesis in lobsters. J. Mar. Biol. Assoc. India.

[52] Nagabhushanam R., Kulkarni G.K. (1981). Effect of exogenous testosterone on the androgenic gland and testis of a marine penaeid prawn, *Parapenaeopsis hardwickii* (Miers) (Crustacea, Decapoda, Penaeidae). Aquaculture.

[53] Maheswarudu G., Rajkumar U., Sreeram M.P., Chakravarty M.S., Sajeev C.K. (2015). Effect of testosterone hormone on performance of male broodstock of black tiger shrimp *Penaeus monodon* Fabricius, 1798. J. Vet. Sci. Photon.

[54] Koskela R.W., Greenwood J.G., Rothlisberg P.C. (1992). The influence of prostaglandin E2 and the steroid hormones, 17α-hydroxyprogesterone and 17β-estradiol on moulting and ovarian development in the tiger prawn, *Penaeus esculentus* Haswell, 1879 (Crustacea: Decapoda). Comp. Biochem. Physiol. Part A.

[55] Fiordelmondo E., Magi G.E., Mariotti F., Bakiu R., Roncarati A. (2020). Improvement of the water quality in rainbow trout farming by means of the feeding type and management over 10 years (2009–2019). Animals.

[56] Dvoretsky A.G., Dvoretsky V.G. (2020). Aquaculture of green sea urchin in the Barents Sea: A brief review of Russian studies. Rev. Aquac..

[57] Samat N.A., Yusoff F.M., Rasdi N.W., Karim M. (2020). Enhancement of live food nutritional status with essential nutrients for improving aquatic animal health: A review. Animals.

[58] Dvoretsky A.G., Dvoretsky V.G. (2012). Does spine removal affect molting process in the king red crab (*Paralithodes camtschaticus*) in the Barents Sea?. Aquaculture.

[59] Dvoretsky A.G., Dvoretsky V.G. (2016). Inter-annual dynamics of the Barents Sea red king crab (*Paralithodes camtschaticus*) stock indices in relation to environmental factors. Polar Sci..

